# Divergent oral cavity motor strategies between healthy elite and dystonic horn players

**DOI:** 10.1186/s40734-015-0027-2

**Published:** 2015-10-21

**Authors:** Peter W. Iltis, Jens Frahm, Dirk Voit, Arun Joseph, Erwin Schoonderwaldt, Eckart Altenmüller

**Affiliations:** Department of Kinesiology, Gordon College, Wenham, MA USA; Biomedizinische NMR Forschungs GmbH am Max-Planck-Institut für biophysikalische Chemie, Göttingen, Germany; Hochshule für Musik, Theater und Medien, Hannover, Germany

**Keywords:** Brass instrument players, Tongue movements, Oral cavity, Real-time (RT) magnetic resonance imaging (MRI), Tongue displacement

## Abstract

**Background:**

This paper describes the use of real-time magnetic resonance imaging in visualizing and quantifying oral cavity motor strategies employed by 6 healthy, elite horn players and 5 horn players with embouchure dystonia.

**Methods:**

Serial images with an acquisition time of 33.3 ms were obtained from each performer during execution of an 11-note harmonic series encompassing 2.5 octaves on a magnetic resonance imaging-compatible horn. A customized MATLAB toolkit was employed for the extraction of line profiles from magnetic resonance imaging films allowing comparative analyses between elite and dystonic horn players.

**Results:**

The data demonstrate differing motor strategies, particularly in moving from the 6th through 9th harmonics. The elite horn player strategy features elevation and anterior displacement of the tongue during ascending sequences, whereas dystonic players showed significantly less movement. The elite horn players thus narrowed the air channel on higher notes, presumably affording faster airflow for vibration of the lips at higher frequencies.

**Conclusions:**

We postulate that failure to employ this strategy by dystonic horn players may require greater tension in the embouchure muscles to compensate for slower air speed. Though this may simply be an expression of or adaptation for dystonia, the possibility that it may be a contributing factor in the development of embouchure dystonia is suggested.

**Electronic supplementary material:**

The online version of this article (doi:10.1186/s40734-015-0027-2) contains supplementary material, which is available to authorized users.

## Background

Focal task-specific dystonia is a movement disorder characterized by the loss of fine motor control which only occurs when executing very specific movement patterns. When it is expressed in the execution of movement patterns required to play a musical instrument, it is often termed musicians’ dystonia [1, 2]. With a reported incidence of about 1 % among professional musicians [3], the etiology of this disorder is complex. It often involves the muscles that have been extensively trained in the finest level of motor control. Repetitive movements requiring high temporal and spatial precision as well as synchronous demands of the musculature seem to be triggering factors. Thus, the expression of dystonic movement in the fingers of guitarists and pianists is common among those affected [1]. Suspected triggers for the development of this disorder include not only the repetition of fine motor activity, but also intrinsic (e.g. genetic predisposition, perfectionism and anxiety) and extrinsic (e.g. complexity of workload-specific movements) factors [4].

Embouchure dystonia is a subcategory of musician’s dystonia affecting the muscles of the lower face, jaw, and tongue which control air flow into the mouthpiece of a wind instrument [5–11]. This painless disorder typically has its onset in the fourth decade, is often restricted to specific technical aspects of playing, may be limited to particular note frequency ranges, and has a variety of phenotypes including lip lock (inability to start notes), tremor, lip pulling (tendency of lips to be drawn out of their normal configuration), jaw lock, and tongue-specific variants [6]. A recent cross-sectional study by Steinmetz et al. [11] suggests that the relative frequency of embouchure-related disorders in a sample of 585 professional brass players was 59 %, resulting in sick leave in 16 % of this population. The prevalence ratios were twice as great in females and in those brass players implementing voluntary mechanical changes in embouchure such as altering mouthpiece placement or lip conformation, or voluntary alterations in breathing technique involving posture and mechanics. The authors suggest that the resulting embouchure disorders (though not embouchure dystonia, per se) may be harbingers of more serious things to come.

Studies of embouchure problems in brass players have been conducted in several ways. Early work focused on descriptive case studies or cross-sectional studies [5, 8, 9, 12], identifying and characterizing various embouchure problems, including embouchure dystonia. Later work specifically targeted embouchure dystonia in an attempt to identify underlying physiologic mechanisms. For example, Hirata et al. [13] compared somatosensory homuncular representations in embouchure dystonia patients and controls and found aberrancies in the dystonic performers suggesting that abnormal somatosensory mapping had occurred. These data were confirmed by subsequent work conducted by Haslinger et al. [14] in which sensorimotor hyperactivity was detected in embouchure dystonia patients. The authors suggest that deficient subcortical and intracortical inhibition accompanied by aberrant sensorimotor integration and reorganization are possible mechanisms. Still other work has attempted to characterize the expression of embouchure dystonia using surface EMG [15] or measurements of tone instability [16].

Despite the suspected involvement of the tongue in embouchure dystonia, there are no published studies that have attempted to describe or quantify activity within the oral cavity in these subjects. The difficulty of imaging dynamic activity inside the mouth during playing is obvious. Conventional radiological techniques, such as computerized axial tomography or magnetic resonance imaging can provide high detail of static positions, but are incapable of assessing dynamic motor activity, and moreover are impractical in assessing movements within the oral cavity during musical performance. Sonography has been utilized to monitor dynamic activity during wind instrument performance [17, 18]. While this method allows some quantitative measures to be obtained (e.g. tongue motion amplitudes), the anatomical resolution provided is somewhat limited, falling short of that provided by MRI.

Recently developed real-time MRI and analysis techniques provide a powerful tool for describing and quantifying dynamic movement patterns of the oral cavity during brass performance. Whether examining discrete snapshots of oral cavity phenomena during movement, slow dynamic movements, or very fast articulatory movements discernable only with 10 msec acquisition rates, the ability to perform accurate quantitative analyses is now possible [19, 20]. It is suggested that such methods may be of use in studying embouchure dystonia. If it is assumed that elite brass performers utilize successful and sustainable motor strategies in executing various performance tasks, then studying this population may provide reference standards against which brass players with dystonia may be compared. Further, if dystonic brass players utilize alternate strategies, it may be possible to draw inferences that contribute to the understanding of embouchure dystonia.

The purpose of the current investigation is to examine these hypotheses by using real-time MRI to characterize, quantify, and compare motor strategies between a sample of elite horn players and a sample of horn players suffering from embouchure dystonia in executing a simple performance task.

## Methods

### Subjects, performance device and testing protocol

Six healthy elite horn players and five horn players diagnosed with embouchure dystonia served as subjects for this study. The elite performers are horn players of international reputation, four currently performing with major U.S. or European symphony orchestras, and two with an active international solo career. The embouchure dystonia players are former professional horn players whose voluntary participation was solicited from a pool of patients who had previously been diagnosed by a movement disorders specialist (author EA) at the Institute of Music Physiology and Musician’s Medicine in Hannover, Germany. The subject characteristics of both groups are documented in Table 1. All testing was performed at the Max-Planck-Institute for Biophysical Chemistry, and prior to participation, all subjects gave written informed consent as approved by the ethics committee of that institution.

**Table 1:** Subject characteristics of elite and dystonic horn players

Gender	Age	Disorder duration (months)	Playing history (years)	Daily practice hours prior to ED	Daily practice hours with ED	Dystonia score
M	60	144	52	3	2	5
M	53	48	43	4.5	1.5	4
M	62	26	53	4.5	3.5	3
M	44	48	35	4.5	1	5
M	45	72	34	4	2	4
M	50	N.A.	48	N.A.	N.A.	N.A.
M	31	N.A.	19	N.A.	N.A.	N.A.
M	63	N.A.	50	N.A.	N.A.	N.A.
F	50	N.A.	35	N.A.	N.A.	N.A.
M	48	N.A.	34	N.A.	N.A.	N.A.
M	57	N.A.	45	N.A.	N.A.	N.A.

Dystonia score key - 5: unable to play the brass instrument due to cramping and dystonic movements, 4: still able to produce sound in certain registers, visible cramping, lip-pull, lip stop, tongue-lock, 3: still able to produce sound in all registers, however sound quality reduced in all registers, subtle visible signs of dystonia such as lip pull and leaks, 2: able to produce sound in all register, however, reduction of sound quality in certain registers, subjective discomfort and cramping, not necessarily visible, 1: professional sound quality, no visible sign of dystonia. (N.A. indicates not applicable)

All subjects performed on a MRI-compatible horn pitched in the key of Eb (Richard Seraphinoff, builder). This horn consists of graded diameter segments of plastic tubing with a plastic mouthpiece at one end, and a non-ferromagnetic brass horn bell at the other end. The horn has no valves, but its acoustical properties allow the performance of an entire harmonic series spanning three octaves. This is an exercise that is commonly practiced by horn players on typical horns, and was famliar to the subjects. The bell was positioned near the feet of the subjects and fixed to the examination table, and the tubing leading to the mouthpiece was extended caudally into the magnet itself so that the subject could play while in the supine position during imaging. Despite the noise generated by the MRI scanner, the subjects were able to hear their playing during all tests, and communication with the investigators was possible due to 2-way intercom system between the control room and the MRI scanner. This also provided a way for recorded examples of each exercise to be played for the subjects prior to their performance.

**Fig. 1:**
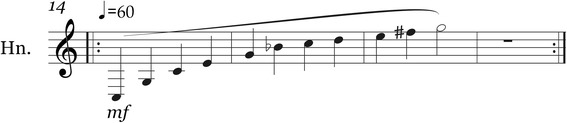
Ascending, slurred harmonic sequence exercise. A single selection from a set of exercises developed for the MRI-horn. Tongued exercise not shown

Two exercises comprised this study: 1) performing a slurred, ascending 11-note harmonic sequence beginning on concert Eb2 and terminating on concert C5 (77.78, 116.54, 155.56, 196, 233.08, 277.18, 311.11, 349.23, 392, 440, and 523.25 Hz, respectively), and 2) performing the same sequence again, but with each note initiated with the tongue. Because the MRI horn has no valves, all note changes were achieved by altering lip tension, air speed, and oral cavity configuration, well-documented strategies typical of horn players [21–23]. The music score for the slurred trials only (horn) is illustrated in Fig. 1. Copies of the exercises were available to the subjects for practice at least three weeks prior to testing. In addition, familiarization with the MRI horn and the exercises was accomplished by allowing 5–10 min of practice outside the scanner. Once in the scanner, practice of individual exercises was also allowed. In both cases, the performers indicated when they felt comfortable and were ready. Each exercise was performed two times, and the trial with the fewest missed notes was chosen for analysis.

### Real-time MRI

All experiments were performed on a 3 T MRI system (Magnetom Prisma, Siemens Healthcare, Erlangen, Germany) using the standard 64-channel head coil. RT-MRI was based on highly undersampled radial FLASH acquisitions with temporally regularized nonlinear inverse (NLINV) reconstruction as previously described [24]. All measurements were performed with an in-plane resolution of 1.5 mm, slice thickness of 10 mm, FOV = 192 × 192 mm^2^ and base resolution 128 × 128 mm^2^. Acquisitions at 30 fps employed the following parameters: TR = 1.96 ms, TE = 1.25 ms, flip angle = 5°, 17 radial spokes per image and *n* = 5 different sets of complementary radial spokes for consecutive acquisitions. Post-processing involved the application of a temporal median filter extending over *n* = 5 frames to reduce residual streaking artifacts and ensure optimum image quality for quantitative analyses. The resulting temporal accuracy of the RT-MRI method has recently been evaluated for small objects moving with velocities of up to 30 cm/s, and movements in the current investigation were well-within the range established for temporal accuracy in previous studies [25].

Online reconstruction and display of real-time images with minimal delay was achieved by a parallelized version of the NLINV algorithm [26] and a bypass computer (sysGen/TYAN Octuple-GPU, 2× Intel Westmere E5620 processor, 48GB RAM, Sysgen, Bremen, Germany) which was fully integrated into the reconstruction pipeline of the commercial MRI system and equipped with two processors (CPUs, SandyBridge E5-2650, Intel, Santa Clara, CA) and 8 graphical processing units (GPUs, GeForce GTX, TITAN, NVIDIA, Santa Clara, CA).

In this study, RT-MRI acquisitions of horn playing tasks were recorded for a period of 30 s corresponding to 900 images. Acoustic recordings relied on a MR-compatible optical microphone (Dual Channel-FOMRI, Optoacoustics, Or Yehuda, Israel) attached to the bell of the French horn, which was placed at about the end of the patient table outside the bore of the magnet. Sound recording was triggered by the radial FLASH sequence and thus synchronized to image acquisition. Further details are provided elsewhere [27].

Prior to conducting data analysis, the audio track for each selected trial was examined using standard audio processing software (Audacity: http://audacity.sourceforge.net/) to determine the moment for each note change. These timings were matched to the exact frame number in the RT-MRI films which then provided an index for determining the number of frames comprising the duration of each note. In this way, it was possible to perform subsequent quantitative measurements during the performance of each note using the custom MATLAB toolbox (RT-MRI toolbox) described later in this paper.

### Data analysis and statistical procedures

**Fig. 2:**
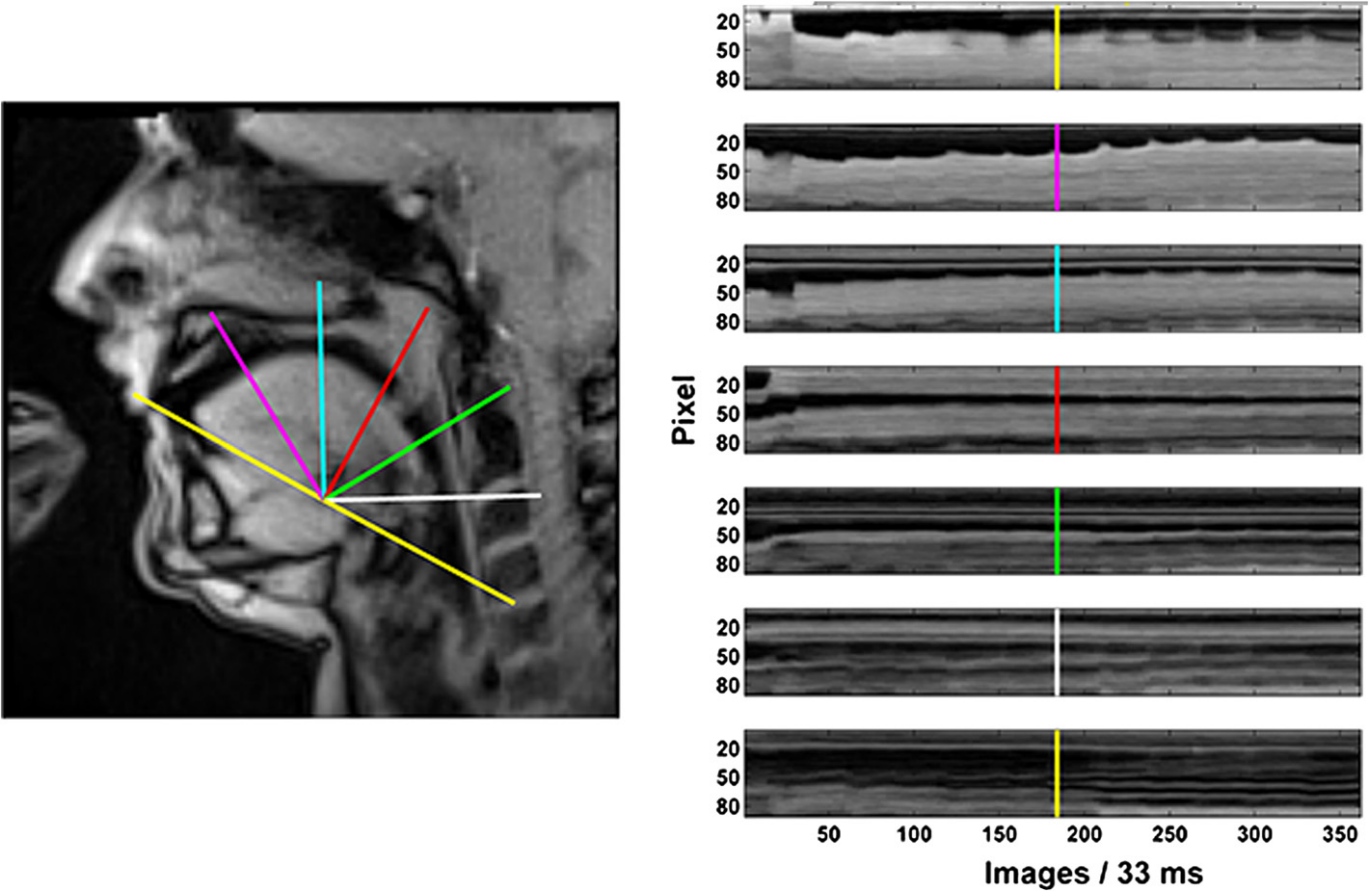
Sagittal view (*left panel*) of an elite horn player at the moment of initiating the 6th note in the harmonic sequence. Seven grid lines are positioned for analysis, and the resulting line profiles (*right panel*) illustrate changes in pixel luminescence along each line during the entire 11-note sequence. Text refers to line profile 2, and the highlighted *vertical marker line* indicates the beginning of the 6th note in the harmonic sequence

The procedures used for obtaining quantitative information from RT-MRI films have been detailed elsewhere [20], so only a brief description will be provided here highlighting unique procedures employed for this study. We utilize a custom RT-MRI toolbox developed for MATLAB (MATLAB R2014a, including the Image Processing and Signal Processing Toolbox) that allows for dynamic data analysis. This toolbox creates a grid over the image of the oral cavity identifying 7 line profiles, each with their own spatial orientation, allowing changes in pixel luminescence across time during each performance task to be studied (see Fig. 2). Within MATLAB, this grid is created by identifying two anatomical landmarks (lower edge of the upper incisor and the anterior edge of the third intervertebral disc) to define a baseline, followed by the automatic creation of an array of 7 segments oriented at 0, 30, 60, 90, 120, 150, and 180° relative to that baseline. Each line was thereby associated with a different region of the oral cavity, thus allowing the study of movements involving different parts of the tongue and throat. Because consistent patterns were seen within the elite subjects in line profile 2 representing the anterior 1/3 of the tongue (second from top, right panel), this line was chosen for all subsequent quantitative analyses comparing the elite to the dystonic performers.

As an example, Fig. 2 and Additional file 1 illustrate an elite performer playing the slurred, ascending 11-note sequence. In line profile 2, vertical undulations appear at the beginning of each note change (for example, frames 97, 124, 154, 184 for the 4th–7th harmonics) and the subsequent baseline between consecutive notes (i.e. the period while each note is sustained) tends to rise with time, particularly during the last half of the exercise. The RT-MRI toolbox allows calculation of the edge position of the anterior-dorsal tongue surface [20], and the precise position of the tongue over time. Thus, for the duration of each note played, the average position of the tongue along the selected line profile was calculated for each subject in each group. Statistical comparisons were made by pooling data within groups, i.e. by calculating the average tongue position on each note within the elite and dystonic performer groups.

The experiment is a repeated measures design having one within-subjects factor (harmonic played, 11 levels) and one between-subjects factor (elite group vs. dystonia group). All statistical tests were executed at the 0.05 significance level. In cases where the assumption of data sphericity was violated, Greenhouse-Geisser adjustments to the degrees of freedom were made to increase the robustness of the analysis.

## Results

**Fig. 3:**
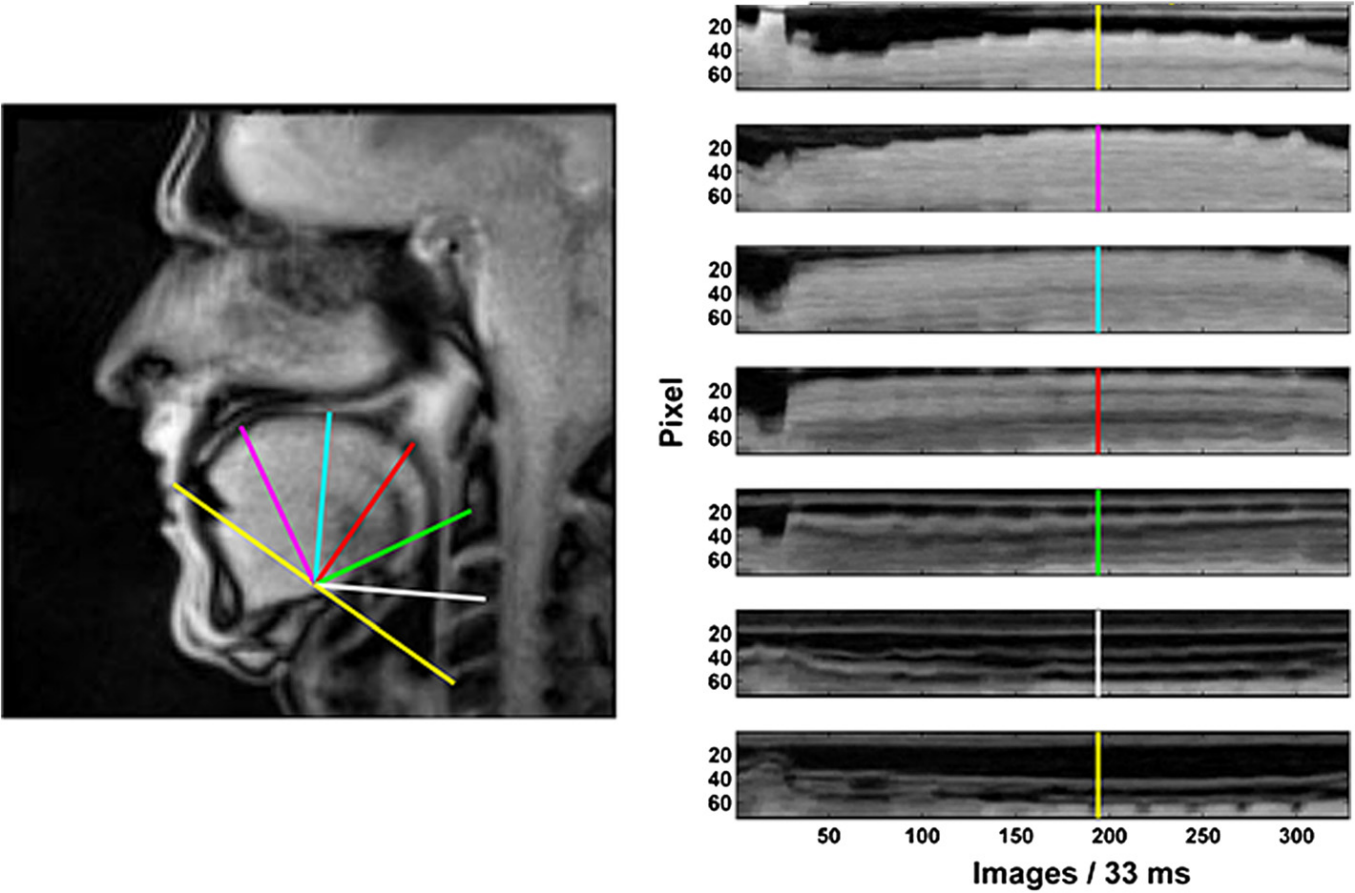
Sagittal view (*left panel*) of a dystonic horn player at the moment of initiating the 6th note in the harmonic sequence. Seven grid lines are positioned for analysis, and the resulting line profiles (*right panel*) illustrate changes in pixel luminescence along each line during the entire 11-note sequence. Text refers to line profile 2, and the highlighted *vertical marker line* indicates the beginning of the 6th note in the harmonic sequence

Visual comparisons of RT-MRI films prior to exercise performance revealed no differences between groups in the resting position of the tongue, teeth, jaw and oral cavity. However, this was not the case during task performance. Figure 3 depicts a sagittal view of one of the dystonic horn players performing the slurred 11-note ascending sequence. Comparing line profile 2 of this performer with that of an elite subject in Fig. 2, there is clear discrepancy in terms of tongue mechanics. While both performers show vertical undulations at the change of each note suggesting tongue elevation, the progressive note-wise elevation of the baseline between inflections identified in Fig. 2 is less evident or absent in the dystonic performer, particularly from the 5th to the 11th harmonics. This observation was typical regardless of whether the notes were slurred or tongued.

For the definition of oral cavitation, a brief referral to line 2 in Fig. 2 (an elite player/slurred sequence) is helpful. In this and all line profile plots, the Y axis has its zero origin position at the top-left, and the vertical length of that axis represents the length of the line in pixels. In this figure, it is about 80 pixels long. The edge created between the oral cavity and the dorsal tongue surface is approximately at the 25 pixel mark in frame 1, drops slightly at the initiation of the first note (Eb2, image 31) rises very slightly over the next 5 notes (6th note, Db4, image 184), and then rises at a greater rate from the 6th through the 9th note (G4, image 268), showing little additional change through the rest of the exercise. Movements toward the origin indicate a decrease in oral cavitation (the tongue moves upward and forward along line 2), while movements away indicate the opposite.

**Fig. 4:**
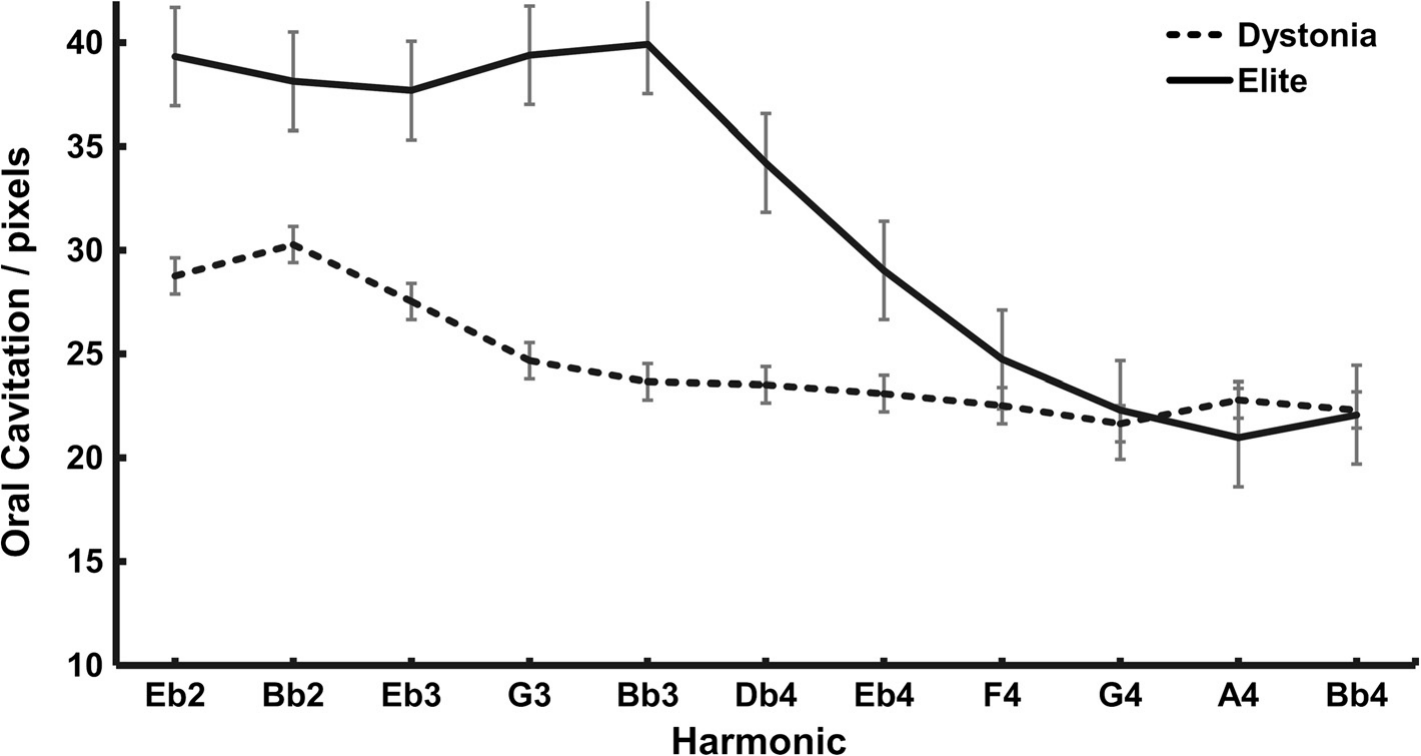
Oral cavitation changes across a slurred 11- note harmonic series. Significant group by harmonic interaction (*p* < 0.001, Greenhouse-Geisser adjusted df)

Figure 4 summarizes these movements for the slurred trials across all subjects by group. In this figure, the height along the Y axis indicates the amount of oral cavitation present during each note. The analysis results in three major findings: 1) in general, the elite players create a larger oral cavity in the lower notes than the dystonic players, 2) both groups tend to reduce the oral cavity moving from low to high notes, and 3) the degree to which the oral cavity is reduced is more pronounced and precipitous in the elite players. Repeated measures ANOVA revealed that the group by harmonic interaction was highly significant (*p* < 0.001 after Greenhouse-Geisser adjustment of the degrees of freedom, observed power = 0.917).

**Fig. 5:**
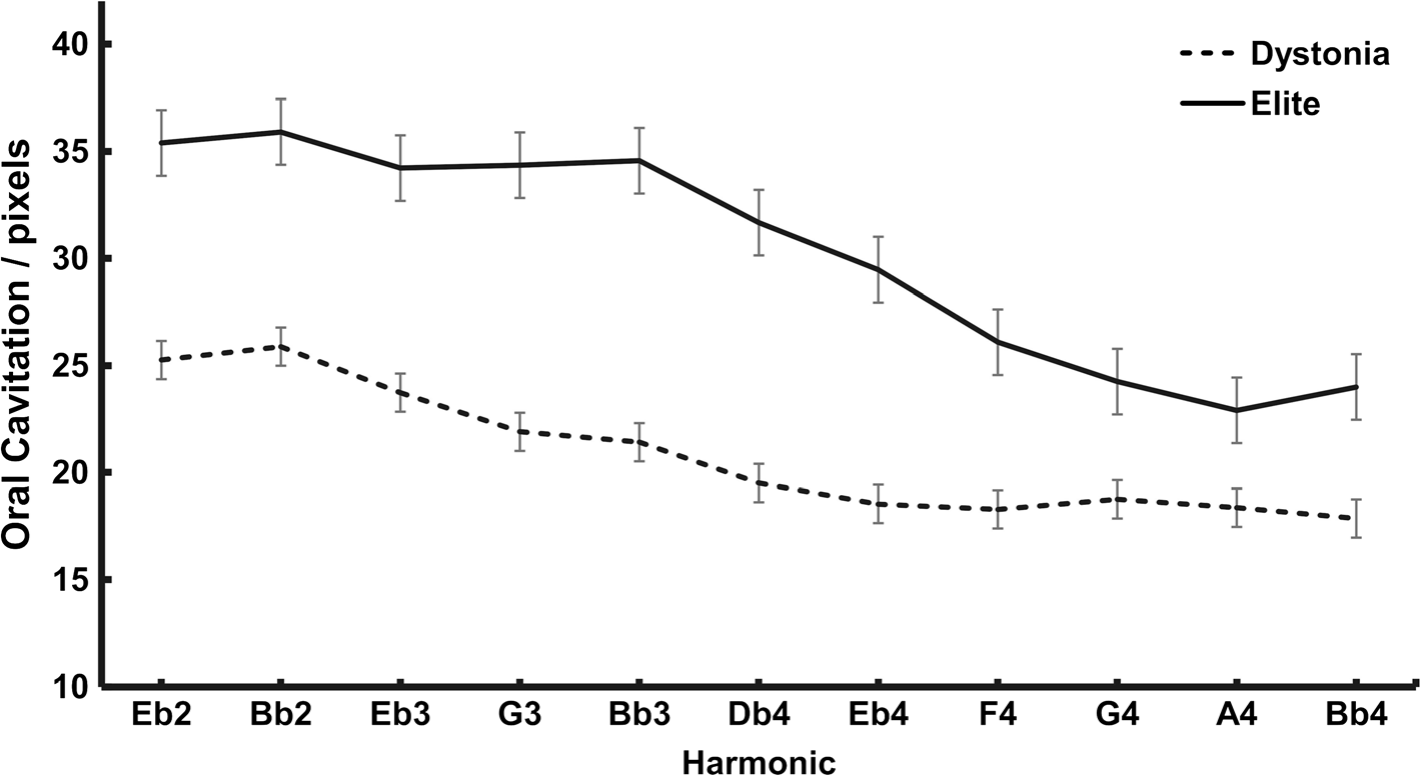
Oral cavitation changes across a tongued 11- note harmonic series. Group main effect is statistically significant (*p* = 0.039, observed power 0.579). No significant group by harmonic interaction

The same tendency was true during the tongued trials, as shown in Fig. 5, though in this case, the repeated measures ANOVA failed to show a significant group by harmonic interaction effect after applying the corresponding Greenhouse-Geisser adjustment of the data (*p* = 0.112, observed power 0.485). Nonetheless, a significant group main effect was demonstrated, with the elite performers having a larger oral cavity than the dystonic players (estimated marginal means of 30.3 and 20.9 pixels for elite and dystonic performers, respectively, *p* = 0.039, observed power, 0.579).

## Discussion

The findings of this investigation suggest that elite horn players utilized a fairly consistent motor strategy across subjects with respect to the anterior-dorsal aspect of the tongue when performing a slurred, ascending 11-note harmonic series. On the lowest notes, the tongue was positioned low within the oral cavity creating a large cavitation until the 5th harmonic was reached. Subsequently higher notes involved a progressive upward and forward movement of the dorsal surface of the tongue that decreased the size of the oral cavity.

These adjustments are similar to those used when phonating the English vowel sounds: ȏ (as in “law”), u (as in “mud”), e (as in “ten”), i (as in “is”), ē (as in “he”). Purposeful use of these vowel/tongue adjustments is advocated by several horn teachers [21, 22, 28]. Among these, the American teacher and artist, Eli Epstein, advocates a systematic association of various notes in the range of the horn to specific vowel/tongue positions, postulating that narrowing the airway results in acceleration of the air column and higher vibration frequencies of the lips. He further suggests that this may reduce excess tension in the muscles supporting the embouchure. Though the degree to which the elite performers adhere to such an approach is not as systematic throughout the range as Epstein recommends, it is apparent that the general pattern is present, particularly in the upper range of the instrument. There, oral cavitation measured along line profile 2 is nearly one-half of that utilized on the lower notes.

In contrast, the dystonic players, though less consistent across subjects, generally display smaller oral cavities on the lower notes, and a less precipitous reduction in cavitation as they move to the higher notes, decreasing oral cavitation by approximately one-third. Though in most cases these performers could execute the appropriate note frequency, their sound was thinner and less stable. Such tonal features are noted by Lee et al. who recently compared tone stability of dystonic players to non-dystonic players [16]. Similar findings have been reported by Iltis and Givens who recorded both audio signals and surface EMG in a dystonic horn player [15]. In that study, both EMG and audio measures showed stochastic patterns in the dystonic horn player indicating marked instability compared to a normal horn player.

The scientific literature to date is limited with regard to describing activity within the mouth during brass performance comparable to the present study. One of the first papers utilizing MRI by Schumacher et al. [29] studied trumpet players, and found that there are concomitant increases in posterior oral cavity area with increasing pitch and loudness. It is noteworthy that these increases were not present in the anterior oral cavity of the performers as these remained small throughout the studied note range. Subsequently, Iltis et al. [20] compared changes in total, anterior, and posterior oral cavity size in performing an ascending 5-note harmonic sequence between a trumpet, horn, trombone, and tuba player. These data suggest that there are between-instrument differences in tongue movements. Specifically, for a trombone and tuba player, the trend was for a decrease in total area as a function of both anterior and posterior oral cavity reduction in moving from low to higher notes. In contrast, the trumpet player showed an increase in total oral cavity area, primarily due to increases in the posterior cavity size. Anterior oral areas were unchanged. In contrast, the horn player (an advanced amateur), showed no change in total, anterior, or posterior oral cavitation during the exercise.

When compared to data in the current study, it is clear that our elite horn players deviate from the horn player described above. This may be explained, at least in part, by the difference in level of expertise. However in addition, the performance devices were considerably different, the former study utilizing a mouthpiece and a plastic resistance attachment (The B.E.R.P., Fairfax, CA) and the current study using an MRI-compatible horn. The MRI-compatible horn mimics an actual instrument and possesses similar acoustical properties. These properties reinforce particular resonant frequencies and provide unique sensory feedback to the player, while pitches performed on the B.E.R.P. are dependent solely on lip tension and air flow, and provide different afferent information. This requires the performer to match pitches without the aid of any the corresponding tactile and acoustical reinforcement properties present in an actual horn.

As mentioned above, the dystonic players do not employ the same tongue movements as the elite performers. They fail to elevate the tongue to the degree shown by the elite players during the ascending exercise, and yet still play the same notes, though with much less stability and tone quality. We propose that in failing to employ the elite strategy, greater tension in the muscles of the embouchure is required in order to increase the vibration frequency of the lips required to play notes of higher frequency. Increased facial muscular tension and co-contraction of non-task-specific muscles is a hallmark of embouchure dystonia [5–8]. This raises the question of whether the increased muscular tension in embouchure dystonia is a compensatory maneuver employed by these players to accommodate a less efficient airway configuration. If it may be assumed that the careers of world-class elite performers are successful and sustainable in part because of employing efficient motor strategies, then it may also be possible that dystonic players habitually use less efficient strategies to compensate for poor technique. Further, the repeated practice of such poor technique may contribute to maladaptive plastic changes in sensory-motor processing [1, 3, 30]. While this idea has some appeal, it must be noted that it is also possible that these less efficient motor strategies are consequences of embouchure dystonia rather than triggers for it. Finally, it must be acknowledged that the expression of symptoms in embouchure dystonia may be seen in structures other than the tongue. Future studies should utilize RT-MRI to study pharyngeal and laryngeal movements in a wider variety of brass and wind instruments, and there is a clear need for obtaining data on larger numbers of subjects.

## Conclusions

We have shown that real-time MRI films can be useful in describing and quantifying movement of the tongue within the oral cavity during performance on an MRI-compatible horn in both elite and dystonic players. Further we have illustrated significant differences in movement strategies between these sample groups that may provide insight into possible triggers for or consequences of embouchure dystonia. Future studies should examine a variety of performance tasks challenging more diverse playing skills in an effort to develop a more complete understanding of this phenomenon. Additionally, studies examining similar comparison groups among different brass instrumentalists (i.e. trumpet, trombone, and tuba) will be useful in extending our understanding.

## Additional file

Additional file 1:
**RT-MRI movie illustrating the sagittal image and representative line profiles of an elite player performing the 11-note ascending harmonic sequence.** (MOV 90310 kb)
